# Probe into the Target and Mechanism of Jianpi Xiaoke Prescription for Treating Type 2 Diabetes Mellitus through miRNA Expression Profiling

**DOI:** 10.1155/2020/7370350

**Published:** 2020-12-15

**Authors:** Qiuyue Guo, Yunsheng Xu, Jie Li, Dan Luo, Jun Li, Cankun Xu, Yanqin Huang

**Affiliations:** ^1^College of Traditional Chinese Medicine, Jingshi Rd. Campus, Shandong University of Traditional Chinese Medicine, Jinan 250014, China; ^2^Department of Endocrinology, Second Affiliated Hospital of Shandong University of Traditional Chinese Medicine, Jinan 250001, China; ^3^First Clinical Medical College of Shandong University of Traditional Chinese Medicine, Jinan 250014, China; ^4^Department of Endocrinology, Affiliated Hospital of Shandong University of Traditional Chinese Medicine, Jinan 250014, China; ^5^Department of Pharmacy, Affiliated Hospital of Shandong University of Traditional Chinese Medicine, Jinan 250014, China

## Abstract

**Methods:**

Ten of the 31 SPF male Wistar rats were randomly taken as the control group; the remaining rats were fed a high-sugar and high-fat diet, combined with Streptozotocin (STZ, 35 mg/kg) that induced a type 2 diabetes model. The model rats were randomly divided into model groups (*n* = 11) and the JPXK group (*n* = 10). After 8 weeks of JPXK intervention, we detected the function of islet cells through HE staining and ELISA. High-pass sequencing technology was adopted to identify the differential expression of miRNA to explore the target of JPXK treatment, assess the relevant target genes, conduct functional analysis, and lastly verify the sequencing data by qRT-PCR.

**Results:**

After treatment, FPG, FINS, and HOMA-IR levels of the treatment group improved significantly compared with those of the control group (*P* < 0.05). Among the miRNAs differentially expressed between the model group and the control group, there were 7 reversals after JPXK treatment, including miR-1-3p, miR-135a-5p, miR-181d-5p, miR-206-3p, miR-215, miR-3473, and miR-547-3p (log2FC ≥ 1 or ≤ −1, *P* < 0.05). Besides, the 1810 target genes associated with these 7 miRNAs were assessed by multiMiR. According to the results of the GO and KEGG analyses, they were associated with biological processes (e.g., glucose transport and fat cell formation), and it covered multiple signaling pathways, capable of regulating islet cell function (e.g., MAPK, PI3K-Akt, Ras, AMPK, and HIF-1 signaling pathways). The PCR verification results were consistent with the sequencing results.

**Conclusion:**

This discovery interpreted the potential therapeutic targets and signaling pathways of JPXK prescription against T2DM based on miRNA expression profiling. In conclusion, our research provided novel research insights into traditional Chinese medicine (TCM) treatment of diabetes.

## 1. Introduction

Type 2 diabetes mellitus refers to a chronic metabolic disease characterized by upregulated blood glucose level and accompanied by symptoms (e.g., insulin resistance, insufficient insulin secretion, and disorders of lipid metabolism), posing serious threats to human health. Statistics suggest that there are nearly 420 million diabetes cases in the world, and this is expected to reach 642 million by 2040 [1]. The pathogenesis of T2DM remains unclear. The medical profession is now actively developing new drugs and seeking feasible supplements to replace drugs.

In recent years, clinical practice and studies have reported that Jianpi Xiaoke (JPXK) prescription, as a traditional Chinese medicine compound, achieved a prominent therapeutic effect on diabetes. JPXK prescription came from the classical “Xiaoke prescription” to treat diabetes in Danxi Xinfa, covering *Astragalus mongholicus*, *Coptis chinensis*, Radix trichosanthis, dried Rehmannia root, Radix cyathulae, and fortune eupatorium herb. Existing studies had reported that JPXK prescription could increase fasting insulin (FINS), reduce insulin resistance (IR), and enhance islet cell function in diabetic rats, as associated with AMPK/mROC1/SAD-A signaling pathway [2]. But we have not yet conducted in-depth research from the perspective of epigenetics.

Epigenetics regulates gene function and expression level mainly through DNA methylation, histone modification, noncoding RNA (e.g., miRNA, lncRNA), and chromatin remodeling. MicroRNAs (miRNAs) refer to endogenous noncoding small RNAs with a length of nearly 21 bases; its acting mechanism is sophisticated, primarily by inhibiting transcription products or degrading target mRNAs to get involved in gene expression [3]. Considerable studies have suggested that miRNAs display a close association with T2DM and its complications [4]. It was reported that miRNAs were highly correlated with islet cell function [5]. After miR-200 expression was upregulated, apoptosis of islet cells was induced by modulating E-box binding to Zeb. Besides, miRNA plays a role in insulin secretion. For instance, miR-375 inhibits the inner reticular structure of the cell membrane by downregulating Mtpn, decelerating the fusion of insulin-containing vesicles with the cell wall, and inhibiting insulin secretion [6]. Overexpression of miR-24 could downregulate the expression of insulin inhibitor Sox6 protein and facilitate the synthesizing and secreting processes of insulin [7].

**Figure 1:**
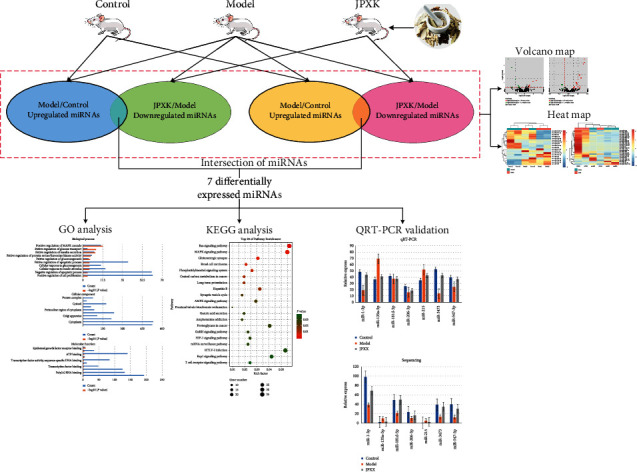
The flow chart of the study. Differentially expressed miRNAs from 2 intersections (Model vs Control and JPXK vs Model). Go and KEGG analyses were used to explore closely related biological roles and signaling pathways. QRT-PCR was used to validate the changes in genes based on the miRNA microarrays.

This study aimed to explore the therapeutic targets and biological processes of JPXK prescription in the treatment of type 2 diabetes through microRNA omics technology. Lastly, the sequencing results were verified by QRT-PCR (Figure 1). Hopefully, this study could provide new treatment insights and research directions for TCM treatment of T2DM.

## 2. Materials and Methods

### 2.1. Materials

The recipe of JPXK prescription includes *Astragalus* 30 g, *Coptis chinensis* 9 g, Radix trichosanthis 12 g, Dried Rehmannia root 12 g, Radix cyathulae 9 g, fortune eupatorium herb 12 g, drugs provided by Jiangyin Tianjiang Pharmaceutical Co., Ltd., decoction concentrated to 1 g/ml, and the specific information of the prescription, as shown in Table 1.

High-sugar and high-fat diet: SPF grade, formula: 10.0% lard, 20.0% sucrose, 10.0% egg yolk powder, 0.5% sodium cholate, as well as 59.5% conventional feed.

  STZ: Sigma, #18883-66-4, America.
  Sodium citrate: Tianjin Zhiyuan Company, #2018010203, China.
  Citric acid: Tianjin Dingshengxin Co., Ltd., #2018-09-16, China.
  Rat insulin (INS) ELISA kit, #CSB-E05070r, China.
  Rat glucagon (GC) ELISA kit, #CSB-E12800r, China.
  RNAprep Pure Cell/Bacteria Kit, Tiangen, China.
  MirVanaTM miRNA Isolation Kit, Qiagen, Germany.
  MiRcute miRNA extraction and isolation kit, Takara, Japan.
  Mir-X miRNA qRT-PCR TB Green Kit, Takara, Japan.

### 2.2. Model Preparation and Grouping of Rats

31 healthy SPF male Wistar rats, weighing (130 ± 10) g, aged 4 weeks, were offered by Beijing Weitong Lihua Company, certificate number: SCXK (Beijing) 2016-0006, attached to the Animal Experimental Center of the Affiliated Hospital of Shandong University of Traditional Chinese Medicine. The rats were adaptively fed for one week with standard feed, ambient temperature 18∼20°C, humidity 60%∼70%, 12 h circadian rhythm, as well as free drinking water. The trial was approved by the Animal Ethics Committee of the Affiliated Hospital of the Shandong University of TCM, with the approval number of AWE-2-19-001.

Ten rats were randomly classified as the control group and fed conventionally, and the remaining rats were fed with a high-sugar and high-fat diet for 8 weeks. Then, the rats fasted for 12 h and weighed and were injected intraperitoneally with Streptozotocin (STZ) (35 mg/kg, dissolved in sodium citrate and citric acid, formulated in 0.1 mmol/L citrate buffer, pH 4.5, at the final concentration of 4%, ice bath, used right after it was ready and used up in 20 min). Rats in the control group were injected with citrate buffer (0.1 mmol/L, pH 4.5) intraperitoneally at a dose of 0.1 ml/100 g. After 72 h, the blood of rats was collected in the tail vein for blood sampling, and fasting blood glucose (FBG) was ascertained. FBG ≥ 16.7 mmol/L for two consecutive times was ascertained for T2DM successfully. 21 rats modeled successfully were randomly classified as the model group (*n* = 10) and the JPXK group (*n* = 11).

According to the equivalent dose conversion formula of humans and animals and the previous research results, the JPXK group was administrated with JPXK decoction (6.46 mg/kg^−1^ d^−1^), which was the optimal concentration of the JPXK. The control group and the model group were given an identical amount of distilled water. Rats in each group were given free access to water for 8 weeks.

### 2.3. Method of Measurement

At the end of the experiment, the rats fasted for 12∼14 h, and subsequently, they were weighed. After anesthesia, the rats were placed in the supine position, and after local disinfection, the abdominal cavity was opened along the midline of the abdomen. 10 ml blood was taken from the abdominal aorta and then centrifuged for 10 min at 3000 r/min. The serum was isolated, and the supernatant was harvested and finally stored in a −80°C refrigerator for subsequent use. The pancreas was removed and placed in a 4% neutral formaldehyde fixative.

#### 2.3.1. Determination of Blood Glucose, Insulin, and Glucagon

  FBG measurement: the FBG was ascertained by Johnson & Johnson's ONETOUCH-Horizon blood glucose meter.
  FINS assay: rat insulin (INS) ELISA kit, strictly consistent with the kit instructions.
  Calculated IR index = FBG × FINS/22.5; insulin sensitivity index (ISI) = 1/(FBG × FINS).
  Serum GC assay: rat glucagon (GC) ELISA kit.

#### 2.3.2. HE Staining of Pancreatic Tissue

The pancreatic tissue of rats was fixed with 10% formaldehyde solution for 12 h, dehydrated by ascending alcohol, and subsequently paraffin-embedded in xylene. The tissue was first condensed into pieces and then cut into 4∼6 *μ*m thick sections. After the tissue was dried at 45°C, HE staining was applied for sealing. Under a microscope, the morphology and distribution of pancreatic islets were characterized.

#### 2.3.3. miRNA High-Throughput Sequencing and Data Acquisition

Total RNA was extracted with the RNAprep Pure Cell/Bacteria Kit from the pancreatic tissue. Lysate RL was prepared according to the kit instructions, the filtrate was collected through filtration column CS, and RNA was extracted through adsorption column R3. The OD value of the quality test RNA reached 260/280 : 1.8∼2.0; the concentration reached ≥500 ng/uL, 28S:18S ≥ 1.5, RIN ≥ 7. After electrophoresis was performed, the small RNA fragments were dephosphorylated, and the 3′ and 5′ end linker sequences were added for reverse transcription and PCR amplification, respectively. The library was prepared and then sequenced on the machine. The sequencing mode was 2 × 150, 20 M reads/sample. The raw sequencing data was identified by the fastx-toolkit, and statistical analysis was conducted. The raw data was quality-dependent with seqtk, while the clean reads were mapped to the reference genome and then transcribed. Under the assistance of Shanghai Sinomics Corporation, the preparation and sequencing of this library were performed. The screening criteria were FC > 2 or <0.5, *P* < 0.05. Firstly, the downregulated miRNA in the model group was screened by comparing the control group, then the upregulated miRNA in the treatment group was screened by comparing the model group, and finally, the intersection was selected. Similarly, the second intersection was obtained, and the total miRNAs obtained from the two intersections were the final result.

#### 2.3.4. miRNA Target Gene Prediction and Functional Analysis

miRNAs were critical to regulating the expression of target genes. Accordingly, multiMiR was adopted in this study for target gene prediction, mainly by searching the 8 databases of DIANA-microT-CDS 5.0, ElMMo 5.0, MicroCosm 5.0, miRanda N/A, miRDB 5.0, PicTar 2.0, PITA 6.0, and TargetScan 7.1 to predict the target genes regulated by differential miRNAs. Besides, functional enrichment analysis of Gene Ontology (GO) and pathway enrichment analysis of the Kyoto Encyclopedia of Genes and Genomes (KEGG) were conducted. The inclusion criteria met *P* < 0.05.

#### 2.3.5. QRT-qPCR

The 7 miRNA therapeutic targets were verified by PCR, the total RNA was extracted using miRcute miRNA extraction and isolation kit, and meantime the purity and concentration were ascertained using a microplate reader. Subsequently, the miRcute enhanced miRNA cDNA first-strand synthesis kit was employed. miRNA first-strand cDNA was reversely transcribed using the A method, and the cDNA was transcribed and then synthesized at 42°C for 60 min at 95°C for 3 min and finally incubated at −20°C. With the use of the miRcute enhanced miRNA fluorescence quantitative detection kit, PCR amplification was performed. The amplification reaction procedure included predenaturation: 95°C for 15 min; PCR reaction: 94°C for 20 s, 60°C for 34 s, for a total of 40 cycles. To verify the sequence data, 7 miRNAs were taken for the QRT-PCR analysis, covering miR-215 (GGACCTATGATTTGACAGACA), miR-135a-5p (CCGGCTTTTTATTCCTATGTGA), miR-206-3p (TGGAATGTAAGGAAGTGTGTGG), miR-181d-5p (CATTCATTGTTGTCGGTGGGT), miR-547-3p (GCGGTAGTTCTTTAAGTGAGA), miR-3473(CTAGGGCTGGAGAGATGGCTA), and mir-1-3p (CGGGAATGTAAAGAAGTGTGTAT). The experimental results were studied using the ΔΔCT method, and the gene expression amount of the model group is denoted as a multiple of the gene expression amount of the JPXK group.

#### 2.3.6. Statistical Analysis

Statistical analysis was conducted using SPSS 21. 0 statistical software. The measurement data are denoted as mean ± SD ($\bar{X}$ ± S). The comparison between groups was drawn by an independent sample *t*-test. The difference was of statistical significance at *P* < 0.05. One-way analysis of variance was conducted to draw a comparison between groups.

## 3. Results

### 3.1. Changes of Blood Glucose before and after Treatment

Compared to the diabetes model, the fasting blood glucose values of the rats in the control group, the model group, and the JPXK group complied with the control range. Before drug intervention, FBG levels in the model and JPXK groups were significantly higher than those in the control group (*P* < 0.05). FBG level after treatment in the JPXK group was significantly lower than that before treatment (*P* < 0.05), and it was noticeably lower than that of the model group at the identical point (*P* < 0.05). More details were in Table 2.

### 3.2. Changes of FINS, GC, ISI, and HOMA-IR

Compared with the control group, the GC and HOMA-IR levels in the model group were remarkably upregulated (*P* < 0.05), and the levels of FINS and ISI were downregulated (*P* < 0.05). After treatment, the levels of GC and HOMA-IR in the JPXK group were downregulated evidently, and the levels of FINS and ISI were elevated (*P* < 0.05). It was revealed that JPXK prescription was capable of facilitating insulin secretion, enhancing insulin resistance, and effectively lowering blood sugar levels (Table 2).

### 3.3. HE Staining of the Pancreas

**Figure 2:**
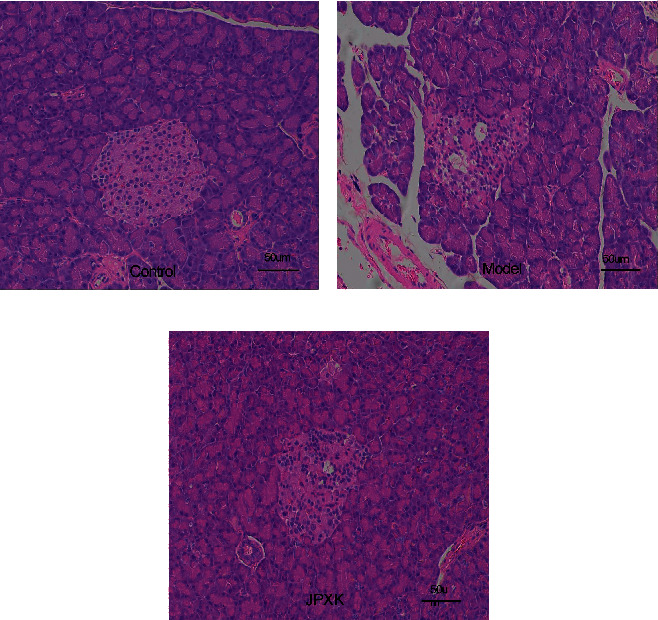
HE staining results of rat pancreatic tissue (10 × 20 times light microscopy). It was obvious that the islet tissue morphology, islet cell morphology, and cell number of the three groups were different.

HE staining result showed that the islets of the control group exhibited regular shape and were scattered in the pancreatic acinar; the islet cells were morphologically regular, clear in boundaries, and arranged neatly, showing numerous numbers; the nucleus was oval and cytoplasm rich (Figure 2).

The model group exhibited irregular islet shape, the islets were atrophied, while some exocrine glands invaded the islets. The islet cells displayed various sizes and shapes, the boundaries were unclear, the arrangement was disordered, and the number was evidently reduced, and some of the nuclei were pyknotic and divided.

The JPXK group exhibited relatively regular and mildly atrophied islet morphology; the islet cell exhibited regular morphology, clear boundary, relatively neat arrangement, and the number more than that of the model group. The number of nuclear light-stained cells increased, and the cytoplasm was abundant in amount.

### 3.4. Screening of Differential miRNA and Therapeutic Targets

**Figure 3:**
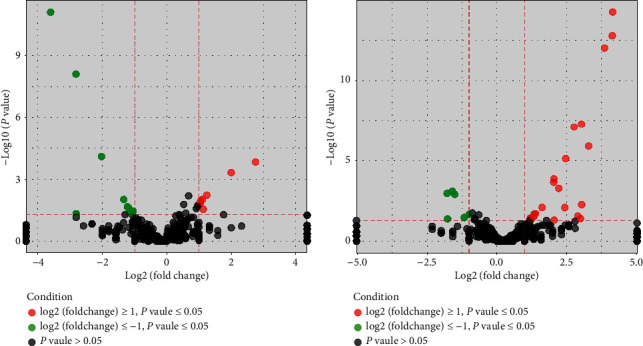
Three groups of miRNA difference representation (volcano map). The dots in the volcano map indicated miRNAs, red for upregulation, green for downregulation, and black for miRNAs that were not eligible for screening. The left panel represented differentially expressed miRNAs in the model group/blank group, and the right panel represented differentially expressed miRNAs in the Chinese medicine group/model group.

**Figure 4:**
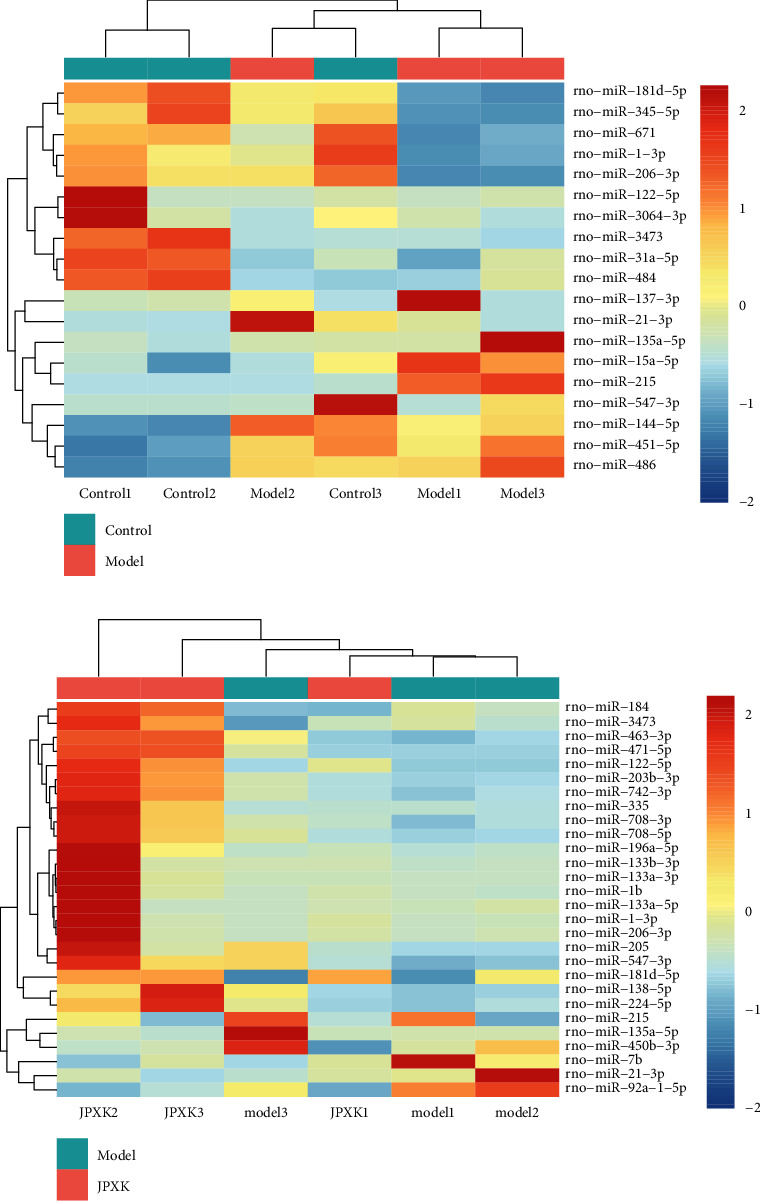
The heat map of miRNA expression. The labels below the cluster heat map represented the sample number, and the one on the right represented the differential miRNAs. K, M, and Z indicated the control, model, and JPXK groups. Each column referred to a sample, and each row denoted a miRNA. White denoted no change in gene level, red indicated upregulation, blue indicated downregulation, and the brightness of color referred to an increase or decrease at the miRNA expression level. The similarly expressed miRNAs were clustered with samples.

Compared with the control group, there were many significant differential miRNAs in the other two groups. The 3 groups of differentially expressed miRNA were visually displayed by the volcano map, as shown in Figure 3, and clustering analysis of these three sets of differential miRNAs is illustrated in Figure 4. According to the screening criteria (log2FC ≥ 1 or ≤ −1, and *P* < 0.05), The expressions of miR-135a-5p and miR-215 were downregulated compared with those before treatment. Compared with the control group, miR-1-3p, miR-181d-5p, miR-206-3p, miR-3473, and miR-547-3p were downregulated in the model group and upregulated in the JPXK group. See Table 3 for details. The results suggested that JPXK prescription regulated the expression of these 7 miRNAs in rat pancreatic tissue to some extent. These miRNAs are the target of treatment for diabetes.

### 3.5. Target miRNA Regulation Gene and Function Analysis

**Figure 5:**
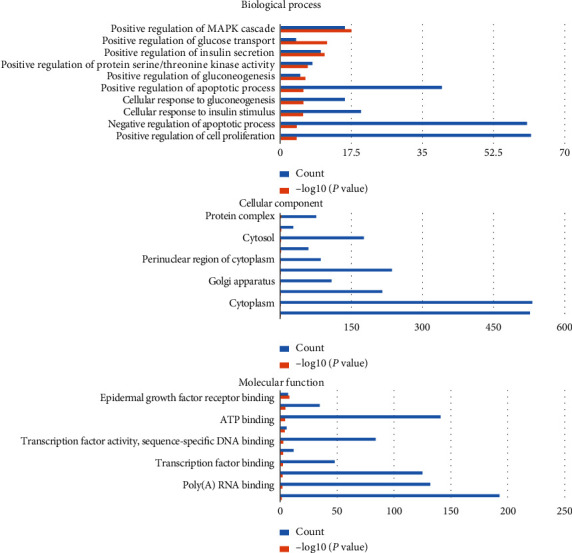
GO Team distribution map of mRNA. As shown in the figure, blue represented the number of enriched targets, and orange represented the −log10 (*P* value). The ordinate indicated the top 10 related biological processes detected, the abscissa indicated the number of enriched targets, and higher columns indicated more targets.

**Figure 6:**
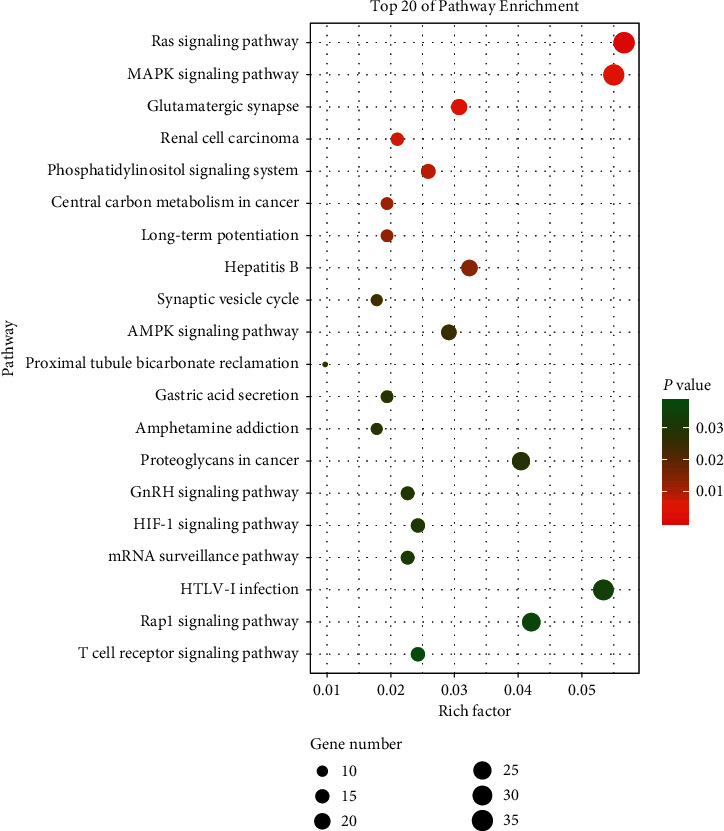
KEGG Signal Path Map of Significant Enrichment of miRNA. The abscissa represents the Rich factor. The larger the Rich factor, the greater the degree of enrichment and the ordinate represents −log10 (*P* value). According to the ranking information of the Rich factor, the important 20 KEGG pathways were displayed.

To identify possible target sites with 1810 relevant genes, e.g., Gpr83, Meox2, Nlrp6, Arf4, Nxt2, Mkln1, and Srsf1, the above 7 miRNA therapeutic targets were analyzed using the software. GO analysis showed that the cell components of the seven miRNA targets were significantly upregulated in the nucleus, cytoplasm, Golgi apparatus, synapses, macromolecular complexes, etc. Their molecular functions were specifically expressed as a protein kinase, phosphoprotein, and transduction molecule activity and participated in biological processes (e.g., glucose transport, adipocyte differentiation, gluconeogenesis, apoptosis, stimulation of insulin, and protein regulation) (Figure 5). KEGG analysis showed that the signal pathway of JPXK prescription in the treatment of type 2 diabetes involves multiple pathways, e.g., MAPK, PI3K-Akt, Ras, and AMPK, HIF-1 signaling pathway, as illustrated in Figure 6. (*P* < 0.05).

### 3.6. The Result of QRT-PCR

**Figure 7:**
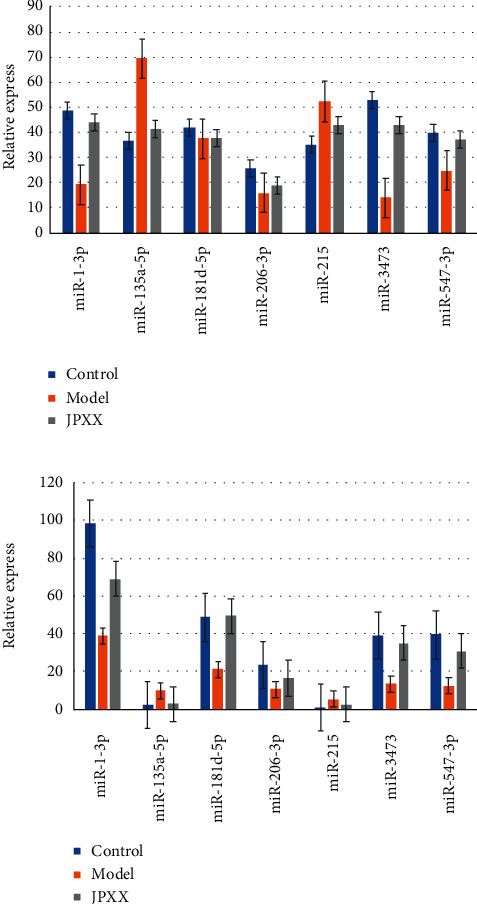
The result of QRT-PCR compared with miRNAseq. In the figure, blue represented the control group, orange represented the model group, and gray represented the JPXK group. The abscissa represented the seven differentially expressed miRNAs, and the ordinate represented the expression of miRNAs in the three groups. The left panel represented qRT-PCR results, and the right panel represented miRNAseq results.

The comparison of the PCR results and the sequencing results suggested that the trends of the differences between the seven therapeutic targets in the model group and the JPXK group were consistent, suggesting that the sequencing results were credible, as presented in Figure 7.

## 4. Discussion

The incidence of type 2 diabetes is high, and the pathogenesis is not fully understood. There have been limited types of therapeutic drugs available in the clinic. JPXK prescription has a good clinical therapeutic effect. Our existing study found that JPXK had the effect of improving insulin secretion deficiency, reducing IR, and effectively lowering blood glucose and was associated with the AMPK signaling pathway [2]. This study once again confirmed the therapeutic effect of JPXK prescription on improving the function of islets.

MiRNAs are endogenous noncoding microRNAs of about 21 to 25 nucleotides in length, which inhibit the translation process or accelerate degradation by binding to target mRNAs, and indirectly regulate the target genes. In this study, seven targets for JPXK prescription treatment of diabetes were found out via miRNA high-throughput omics technology, which were miR-215, miR-135a-5p, miR-1-3p, miR-181d-5p, miR-206-3p, miR-3473, and miR-547-3p. These miRNAs might be associated with the onset of type 2 diabetes and decreased islet cell function. JPXK prescription could change the expression of these miRNAs and make them incline to the control group, indicating that JPXK prescription reduced FBG and improved the function of pancreatic islet cells that may be related to these miRNAs.

According to reports in the literature, the expression profiles of the above seven therapeutic targets are closely associated with islet cell function, adipogenesis and differentiation, cell senescence, and lipid metabolism. For instance, miR-3473b inhibits macrophage activation and inflammatory response by targeting phosphatase and angiotensin homolog (PTEN), promoting Akt/GSK-3 signal transduction and IL-10 production [9]. Upregulation of miR-3473b gene expression and high-efficiency inhibition of macrophage activation increased serum anti-inflammatory cytokine IL-10 levels in diabetic rats and effectively improved insulin resistance and chronic inflammatory response in diabetic patients [10]. Nie J et al. found that miR-215 targets the mammalian target of rapamycin (mTOR), and overexpression of miR-215 can significantly downregulate the expression of mTOR which was an important protein in the insulin signaling pathway and acts as a two-way regulator between AMPK and SAD-A [11]. Our existing studies have found that JPXK prescription can improve islet cell function, regulate insulin secretion, and correlate with AMPK/mTOR/SAD-A signaling pathway [2]. In connection with this study, it is speculated that this gene regulates mir-215 gene expression and targets regulation mTOR and bidirectional regulation of AMPK and SAD-A, maybe the mechanism by which JPXK prescription can improve the function of islet cells and treat diabetes through AMPK/mTOR/SAD-A signaling pathway [12]. Other studies have shown that miR-206-3p, miR-215, and miR-135a participate in the formation of adipocytes. miR-215 and miR-135a activate the Wnt/*β*-catenin signaling pathway by targeting related proteins, thus hindering triglyceride accumulation and adipogenesis and differentiation [13, 14]. miR-206-3p inactivates PI3K/Akt signaling pathway by silencing c-Met protein, inhibits the formation of fat cells, and then adversely affects the conversion of glucose into fat [15]. According to the results of GO and KEGG analysis, the therapeutic pathway was obviously associated with the PI3K/Akt signaling pathway, and it was speculated that JPXK prescription activates the PI3K/Akt signaling pathway by regulating target genes. Also, target gene predictions indicate that mir-1-3p is associated with Arf4, and in 2017, Pearling et al. found that mir-1-3p could target Arf4 expression and activate the PCK pathway, regulate insulin secretion, and improve insulin resistance [16], which was consistent with the conclusion of this study. Several studies had indicated that the mechanism of action of JPXK prescription against diabetes might also be associated with its enhancement of endothelial cell function. Upregulation of lncRNA NEAT1 expression activated the miR-181d-5p/CDKN3 functional axis [17]. Downregulation of miR-1-3p expression inhibits endothelin-1 (ET-1) activity, inhibits vascular endothelial cell injury, and improves dysfunction [18]. Besides, the activity of ET-1 was enhanced in a high glucose environment, indicating that the increase of blood glucose inhibited the expression of mir-1-3p, which was consistent with the downregulation of mir-1-3p in the model group. The accuracy of the experimental results were confirmed.

Functional analysis suggested that multiple pathways display close association with insulin function decline, insulin secretion, and glycolipid metabolism (e.g., MAPK, PI3K-Akt, Ras and AMPK, HIF-1, and insulin signaling pathway). Several studies had suggested that diabetic patients have impaired AMPK activity [19]. Existing studies had reported that JPXK prescription could facilitate glucose absorption by inhibiting the absorption of glucose by activating the AMPK pathway and hindering glucose production [2]. Drugs used to treat diabetes (e.g., metformin) also act by modulating AMPK [20]. Besides, Chen et al. detected that berberine BBR affected mitochondrial function by activating AMPK and PI3K-Akt-eNOS signaling pathways in diabetic rats and exerted antiapoptotic effects, thereby promoting the recovery of functions of pancreatic islets and cardiomyocytes caused by diabetes [21]. Zhu et al. also reported that Roxadustat accelerates wound angiogenesis and promotes wound healing in diabetic skin by activating the HIF-1/VEGF/VEGFR2 pathway [22]. Research had suggested that the concentration of serum HIF-1*α* in type 2 diabetic patients was remarkably higher than that in the control group. Accordingly, it is speculated that HIF-1*α* may be involved in the process of inflammation, angiogenesis, and vascular endothelial injury in diabetes [23]. KEGG analysis also suggested that the way of JPXK prescription in the treatment of diabetes was also associated with RAP 1. The result was consistent with other studies that RAP 1 was closely associated with lipid metabolism, inflammation, and oxidative stress, and the specific mechanism requires subsequent study [24]. Our studies revealed that JPXK prescription had multiple pathways and links to improve the function of islet cells in T2DM rats, increase insulin secretion, reduce FBG, and mitigate cell apoptosis.

## 5. Conclusion

Our study discovered the 7 therapeutic targets and biological processes of JPXK prescription in the treatment of type 2 diabetes through microRNA omics technology. In the subsequent experiment, we will continue to study the related mechanisms of these seven miRNAs through knocking out, silencing, and overexpressing these related miRNAs to provide new research insights and treatment directions for TCM treatment of diabetes.

## Figures and Tables

**Table 1:** Components of JPXK (herbs were combined in a 30 : 9 : 12 : 12 : 9 : 12 ratio).

Latin name	Family	English name	Chinese name	Part used
Radix astragali	Leguminosae	*Astragalus mongholicus*	Huang Qi	Root
Rhizoma coptidis	Ranunculaceae	*Coptis chinensis*	Huang lian	Root
Radix trichosanthis	Cucurbitaceae	Radix trichosanthis	Tian huafen	Root
Radix Rehmanniae	Scrophulariaceae	Dried Rehmannia root	Sheng Di Huang	Root
Radix cyathulae	Amaranthaceae	Radix cyathulae	Chuan Niu Xi	Root
Herba eupatorii	Compositae	Fortune eupatorium herb	Pei lan	Stem and leaf

**Table 2:** Comparison of FGB, FINS, GC, HOMA-IR, and ISI levels in each group of rats.

Group	Number	FBG (mmol/L)		FINS (mIU/L)	GC (pg/L)	IR	ISI
		Before treatment	After treatment				
Control	10	4.31 ± 0.25	4.25 ± 0.79^△^	20.12 ± 0.15	154.73 ± 12.55	5.13 ± 0.96	3.75 ± 1.89
Model	10	28. 67 ± 2. 49	27. 85 ± 3.07^∗^	13.58 ± 2.21^∗∗^	230. 11 ± 17. 64^∗∗^	18. 06 ± 0. 92^∗∗^	8.02 ± 2.71^∗∗^
JPXK	10	27. 95 ± 3. 11	20. 27 ± 1.36^#^	16.17 ± 1.06^△△^	201.50 ± 18. 21	15. 62 ± 1. 37^△△^	7.29 ± 1.36

^#^ *P* < 0.05 vs the predose group of this group, ^Δ^*P* < 0.05 vs the control group, ^*∗*^*P* < 0.05 vs the model group, ^*∗∗*^*P* < 0.05 vs the control group, ^ΔΔ^*P* < 0.05 vs the model group.

**Table 3:** Comparison of 7 predicted therapeutic targets (log2FC ≥ 1 or ≤ −1, and *P* < 0.05).

Gene name	Family	MIR	Model/control		JPXK/model	
			Log2FC	*P* value	Log2FC	*P* value
miR-3473	No [8]	MIMAT0024853	−3.37	0.000	1.39	0.019
miR-547-3p	mir-547	MIMAT0012851	−1.68	0.002	1.32	0.030
miR-1-3p	mir-1	MIMAT0003125	−1.33	0.018	4.15	0.000
miR-181d-5p	mir-181	MIMAT0005299	−1.19	0.044	1.21	0.036
miR-206-3p	mir-1	MIMAT0000879	−1.15	0.049	4.14	0.000
miR-135a-5p	mir-135	MIMAT0000841	2.00	0.003	−1.79	0.001
miR-215	mir-192	MIMAT0003118	2.58	0.002	−1.17	0.033

## Data Availability

The original data used to support the findings of this study are included within the article.
